# Islet transplantation: overcoming the organ shortage

**DOI:** 10.1186/s13098-023-01089-8

**Published:** 2023-07-01

**Authors:** Marluce da Cunha Mantovani, Ilana Gabanyi, Carlos Andrés Pantanali, Vinícius Rocha Santos, Maria Lúcia Cardillo Corrêa-Giannella, Mari Cleide Sogayar

**Affiliations:** 1 Cell and Molecular Therapy NUCEL Group, School of Medicine, University of São Paulo, Avenida Dr. Arnaldo, 455, São Paulo, 01246-903 SP Brasil; 2 Technical Division for Teaching, Research and Innovation Support - DTAPEPI Biotechnology and Innovation Facility, School of Medicine, University of São Paulo Medical School, São Paulo, 01246-903 SP Brazil; 3 Gastroenterology Department, School of Medicine, University of São Paulo, São Paulo, 01246-903 SP Brazil; 4 Medical Sciences Department, Laboratory of Carbohydrates and Radioimmunoassay (LIM-18) HCFMUSP, Medical School, University of São Paulo, São Paulo, 01246-903 SP Brazil; 5 Biochemistry Department, Chemistry Institute, University of São Paulo, São Paulo, 05508-000 Brazil

**Keywords:** Organ donation, Diabetes *mellitus*, Pancreas, Islet transplantation, Pancreas donors profile and selection

## Abstract

**Background:**

Type 1 diabetes *mellitus* (T1D) is a condition resulting from autoimmune destruction of pancreatic β cells, leading patients to require lifelong insulin therapy, which, most often, does not avoid the most common complications of this disease. Transplantation of isolated pancreatic islets from heart-beating organ donors is a promising alternative treatment for T1D, however, this approach is severely limited by the shortage of pancreata maintained under adequate conditions.

**Methods:**

In order to analyze whether and how this problem could be overcome, we undertook a retrospective study from January 2007 to January 2010, evaluating the profile of brain-dead human pancreas donors offered to our Cell and Molecular Therapy NUCEL Center (www.usp.br/nucel) and the basis for organ refusal.

**Results:**

During this time period, 558 pancreata were offered by the São Paulo State Transplantation Central, 512 of which were refused and 46 were accepted for islet isolation and transplantation. Due to the elevated number of refused organs, we decided to analyze the main reasons for refusal in order to evaluate the possibility of improving the organ acceptance rate. The data indicate that hyperglycemia, technical issues, age, positive serology and hyperamylasemia are the top five main causes for declination of a pancreas offer.

**Conclusions:**

This study underlines the main reasons to decline a pancreas offer in Sao Paulo—Brazil and provides some guidance to ameliorate the rate of eligible pancreas donors, aiming at improving the islet isolation and transplantation outcome.

*Trial registration*: Protocol CAPPesq number 0742/02/CONEP 9230.

## Background

Human islet isolation and transplantation has been performed in several Centers around the World over the past 20 years [1–5]. Islet transplanted type 1 diabetes *mellitus* (T1D) patients present marked improvement in both short-term and long-term outcomes, with insulin independence after initial or subsequent transplantation being achieved in up to 80% of the patients one year post-transplantation [6]. Approximately 50% of the patients remained insulin-independent at five years after receiving the transplant. Islet transplantation is already approved and reimbursable by insurance companies or covered by National Health Systems in several countries, including Canada, Australia, the United Kingdom, Switzerland, Italy, France and other parts of Europe, in total of 40 countries approximately [3, 5]. Over 2,800 procedures have been performed worldwide in 1,399 recipients [7]. In spite of being a promising treatment for patients with brittle T1D displaying hypoglycemia unawareness, it is severely limited, among other factors, by the shortage of organ donors maintained under adequate conditions.

The multidisciplinary team of our Cell and Molecular Therapy Center (NUCEL) derives from a basic research group focused on control of cell proliferation and differentiation, in close association with clinicians and surgeons from local hospitals. Clinical cell therapy for T1D patients and production of biopharmaceuticals for both human and animal use have been the focal points of interest of this biotechnological Center, which has been operating, at the University of São Paulo, since the early 2000 [8]. As the first Center for human islet isolation and transplantation in Brazil [9], we are constantly seeking for means to obtain a good yield of high quality islets to increase the possibility of offering this treatment to a larger number of patients.

During the period of 2007 – 2010, our Center received a large number of pancreas offers, however, only a few of them could be accepted, in accordance with our inclusion and exclusion criteria. Therefore, we decided to investigate the profile of our candidate pancreata donors, taking into account the most important refusal causes, in order to initiate a search for effective actions to increase the proportion of effective donors, which could allow improving the acceptance rate and to discuss the process of pancreatic islets donation and transplantation in Brazil.

## Methods

### Study design

In this study, we retrospectively analyzed the profile of multiple organ donors whose pancreata were offered to our NUCEL Center for human islet isolation and transplantation from January 2007 to January 2010, period of a large number of multiple organ donors profiles offered to our team. Human pancreata from adult heart-beating donors were harvested in accordance with Brazilian regulations and the local Institutional Ethical Committee (Protocol CAPPesq number 0742/02 / CONEP 9230). Criteria for acceptance or refusal of pancreata donors for islet isolation based on international criteria from worldwide islet transplantation reference centers were established in our laboratory [10] as follows: age below 18 above 60 years old, drug addiction, ethylism, infeccious diseases, seropositivity for Chagas disease, toxoplasmosis, rubella, syphilis, cytomegalovirus, hepatitis B and C and human immunodeficiency virus (HIV), neoplasia, hemodilution, history of diabetes, cold ischemia time of more than 12 h. The following criteria were also used, but exclusion was decided on a case-by-case basis: Intensive Care Unit (ICU) stay greater than nine days, amylase above 400 U/L, persistent hyperglycemia (200 mg/dL), cardiorespiratory arrest, serum creatinine 50% higher than the initial value, hemoglobin < 6.0 g/dL, unstable donor and macroscopic aspect of the pancreas. All data from organ donors were collected from those available in the form named “Information about the multiple organ donor” of the Brazilian Federal Government Transplantation Center (TC) (https://www.gov.br/saude/pt-br/composicao/saes/snt), which is responsible for coordinating the entire transplantation system in the State of São Paulo. This form summarizes all general data referring to the organs or tissues subject to transplantation. All data available on organ donors were catalogued and classified according to the exclusion criteria. Each organ refusal was due to up to four different reasons, which were independently analyzed and taken into account in our analysis. Acceptable values of glycemia are between 60 and 200 mg/dL and of amylase are < 400 U/L.

## Results

From January 2007 to January 2010, 558 organs (pancreas) from heart-beating multi-organ donors were rejected by whole organ pancreas transplantation teams, being then offered by the State of São Paulo Transplantation Center for islet isolation. Considering the exclusion criteria, 512 pancreas were refused and 46 were accepted. Despite this seemingly adequate donor pool, only approximately 10% of the potential donors became actual donors.

In order to define the main obstacles for organ acceptance, we determined the profile of potential donors and listed the refusal causes for each possible pancreas donor (Table 1). The profile showed that the gender distribution of the potential donors was similar. The body mass index (BMI) average was 25.7 kg/m^2^. With respect to the age range, we observed that 59% of the donors were between 40 and 60 years old (average 45.8). Most of our potential donors were normoglycemic and presented normal amylasemia.

**Table 1:** Profile of the pancreata donors offered for islet transplantation with respect to gender distribution, age, glycemia and amylasemia

Gender %	
Male	54
Female	46
Age (years old)	
< 25	13
26—39	13
40—50	27
51—60	31
> 60	16
Glycemia	
Normal (60–200 mg/dL)	67
Altered (> 200 mg/dL)	33
Amylasemia	
Normal (< 400 U/L)	74
Altered (> 400 U/L)	26

Data are expressed in percentage. (n = 558 corresponding to all the potential donors from 2007 to 2010)

We found that the main cause for rejecting a pancreas offered for islet transplantation was hyperglycemia (14.8%), followed by technical issues (8.7%), age (8.5%), positive serology (8.4%) and hyperamylasemia (8.2%) (Table 2).

**Table 2:** Classification according to occurrences and main reasons to refuse a pancreas offered for islet isolation and transplantation

	%
Hyperglycemia	14.8
Technical issues	8.7
Age	8.5
Seropositivity for Toxoplasmosis, Rubella, Syphilis, Cytomegalovirus, Chagas, Hepatitis B and C, and HIV	8.4
Hyperamylasemia	8.2
Alcoholism	7.0
Abnormal laboratorial exams	6.6
Cardio respiratory arrest	6.6
Prolonged ICU stay	5.3
Systemic hypertension	4.7
Unstable donor	4.4
Infection	4.4
Diabetes mellitus	3.3
BMI < 24	3.3
Unsatisfactory macroscopic pancreas aspect	2.8
Presence of tumor	1.1
Cold ischemia > 8 h	1.0
Drug abuse	0.9

Data are expressed in percentage. (n = 558 corresponding to all the potential donors from 2007 to 2010). BMI: Body Mass Index, ICU: Intensive Care Unit

With respect to the technical issues, detailed in Table 3, our group considered as the main causes for pancreas refusal unavailability of qualified personnel for processing, problems related with availability of materials and equipment and difficulties in organ procurement due to transportation over long distances.

**Table 3:** Distribution according to occurrence of technical issues leading to refusal of a pancreas offered for islet isolation and transplantation

Technical issues	%
Unavailability of qualified personnel for processing	34.5
Difficulties in organ procurement due to long transportation distance	34.5
Material unavailability	31.0

## Discussion

In Brazil, during 2022 (January – September report) organ donation has reached the figure of 16.4 per million (pmp) organ effective donors [11]. The last report of The Brazilian Association for Organ Transplant (ABTO) showed that the rate of organ donation increased in the last eight years (from 49,0 pmp in 2014 to 57,7 pmp in 2021), the actual number of effective donors being 14,2 pmp in 2014 to 15,1 pmp in 2021) [12], leading us to believe that further improvements must be implemented to improve the rate of effective donors.

The Brazilian National Transplantation System (NTS) is regulated by Federal Law 9434, dated February 4th, 1997 and Federal Decree number 2268, dated June 30th, 1997. The NUCEL Center is located in São Paulo, the largest city of the State of São Paulo, of approximately 46 million people, with the notification rate of potential donors reaching 72.4 pmp in 2022, 71.6% of which were refused [11]. In August 13th, 2010 Resolution SS 151 was created, stemming from the need to update the standards enforced in the State of São Paulo, with respect to the operation of the State Transplantation System – SST [13]. This Resolution reduced the number of organ donations for pancreatic islet transplantation, since the families of deceased donors were required to choose between donating the organs only for transplantation or for both transplantation and scientific/medical research.

In 2011, a major milestone was the publication of the Resolution of The Collegiate Board (RDC) No. 9, dated March 14th, by the Brazilian Regulatory Agency (ANVISA) [14]. Minimum technical and sanitary requirements for Technological Cellular Centers operation were established and, also, rules for collection, processing, packaging, storage, quality control evaluation, disposal and approval for release of human cells and their derivatives were published, contemplating clinical research and stem cell therapy. This resolution was subsequently amended by RDC No. 214 (February 7th, 2018) [15]. Also in 2011, ANVISA published RDC No. 23 (May 27th), which establishes the technical regulation for operation of germinative tissue and cell biobanks and provides other regulations [16]. This resolution was subsequently amended by RDC No. 72 (March 30th, 2016) [17].

The first pancreatic islet transplantation in Brazil was performed by our group in 2002 in a T1D patient with recurrent severe hypoglycemia and metabolic instability [9]. This was possible due to the effort of a multidisciplinary group, including a team of specialized surgeons and clinicians, in addition to our Cell Biology team, which is responsible for islet isolation, purification and quality control. Our Islet Unit complies with all requirements for Current Good Manufacturing Practices—cGMP facilities for isolation and purification of human pancreatic islets. Our NUCEL group was responsible for treatment of five patients, totalizing 11 islet infusions, resulting in clear benefits upon transplantation, namely: decreased or elimination of hypoglycemic episodes, lower requirement for exogenous insulin administration, production of detectable levels of C-peptide and absence of hyperlability.

### Hyperglicemia

Elevated glycemia, which occurred in 14.8% of the cases, was the first cause of pancreas refusal for islet transplantation. Considering that glycemia influences the general state of the endocrine pancreas, we investigated what could cause hyperglycemia in order to evaluate whether it would be possible to better control these variables and improve the quality of the pancreas offered for islet transplantation.

It is known that hyperglycemia can be harmful to the pancreatic islets, since it is associated with increased rate of β-cell death [18]. Therefore, the pancreas of a hyperglycemic donor is less likely to result in a good yield of adequate islets for transplantation. This is one of the reasons why we refuse pancreas from a donor displaying hyperglycemia (glycemia > 200 mg/dL). Also, following the 2022 guidance of the Brazilian Diabetes Society (SBD) [19], the diagnosis of diabetes should be made using a random blood glycemia, measured at any time of the day, independently of the meal periods, should be > 200 mg/dL, in the presence of unequivocal symptoms of hyperglycemia. However, since potential donors are maintained at the ICU, most of the time receiving glycosylated serum and parenteral nutrition, it is impossible to measure their glycemia level in fast, therefore, we considered, as normal glycemia, values between 60 and 200 mg/dL.

When a patient is maintained at the ICU, several factors may account for unstable glycemic levels. Some medications, such as corticosteroids and thiazide diuretics, can elevate glycemia [20], while some compounds, such as dopamine, may induce a high insulin secretion, which can also compromise β-cells function and viability [21]. Moreover, continuous use of glycosylated serum, without strict control, may generate hyperglycemia in the possible donor, generally aggravated with a prolonged stay at the ICU. Therefore, we believe that better management of the donor at the ICU could avoid several cases of hyperglycemia, decreasing not only the number of refused pancreas due to the glycemic levels, but, also, in cases of patients considered as unstable (alternating between hypo- and hyperglycemia associated with other limiting values ​​of pancreata refusal criteria). These unstable patients are responsible for 4.4% of the refusal cases in our study.

Another important issue related to the glycemic levels is the capacity to determine whether the potential donor has diabetes. In our study, 3.3% of the refusal cases were due to the presence of diabetes in the donor. We believe that this number is underestimated because the tests performed are not adequate to verify whether or not the donor is diabetic, since they were only based on information provided by the patients’ family members and on glycemic levels, both of which are not reliable sources. Inclusion of a glycated hemoglobin test, which represents an assessment of the average plasma glucose over the past six to eight weeks [22], in the laboratorial exam routine for potential donors would be a valuable information for the teams interested in receiving pancreas for islet transplantation, enabling a more objective selection of the offered organs.

### Technical issues

The second cause of pancreas refusal for islet transplantation was technical issues (8.7%). In Brazil, when a possible heart-beating donor is available, the hospital notifies the Transplantation Center (TC), which contacts the Organ and Tissues Procurement Organization Service (SPOTs), which then contact the local hospital to verify whether the patient can become an organ donor, and, as soon as the donation is confirmed, the SPOTs contact the TC, whose teams search for probable receptors and call the transplantation teams. Specifically, for islets isolation, a medical pancreas harvesting team goes to the hospital in order to remove the organ and send it to our laboratory, where the islets are isolated in a Bioclean Room under cGMP facilities.

The effectiveness of the islet isolation procedure is given by the yield in terms of iEQs (islet equivalents based on an islet of 150 µm diameter, thereby mathematically compensating for the islet volume). In a previous study of our group [10] we adopted as a good outcome for islet isolation yields the value of 200,000 IEQs, an intermediate number when compared with those found in the literature (Lakey and cols. [23] and Kim and cols. [24] defined 100,000 IEQs, while Hanley and cols. [25] adopted 250,000 IEQs and Matsumoto and cols. [26] used 300,000 IEQs as a reference for a good islet isolation outcome). In the present study, we adopted yields of 100,000 IEQs as being satisfactory because during this period only 10% of the donors offered displayed BMI > 30 kg/m^2^ and our previous study showed that pancreata from donors with BMI > 30 kg/m^2^ yielded an average of 277,964 IEQs, while organs from donors with BMI < 30 kg/m^2^ produced 104,874 IEQs (p < 0.001) [10]. In general, 68% of the pancreas accepted by our group resulted in yields of > 100.000 iEQs. However, the yield of each islet preparation used to infuse into those five patients was greater than 300,000 IEQs, the remaining ones being used for research only.

After a rigorous quality control procedure, the islets may be infused into T1D patients displaying metabolic instability and unaware hypoglycemia, being carefully enrolled by a team of endocrinologists.

Due to this multifactorial, laborious and expensive process, technical issues were the second major cause for pancreas refusal. These difficulties include: insufficient number of qualified personnel, problems related to availability of specific reagents and supplies and problems with organ transportation due to large distances between the hospital where the donors are being maintained and the TC.

Organ procurement is a crucial component of the islets isolation and transplantation process. Due to the high costs of maintaining organ donors at ICUs in Brazil, there is a shortage of deceased organ donors, which can make it difficult to provide transplantation services to all those who need them. In order to overcome, or at least diminish, these issues, some actions may be taken, such as, for example, increasing the number of pancreata procurement teams to allow for more efficient and timely procurement of donor organs. This could help to increase the supply of organs available for pancreatic islet transplantation and research. Another possible solution is to increase the number of pancreata procurement teams specialized in islet transplantation in different regions of the country. This could help to shorten the distances that patients need to travel, potentially reducing costs and increasing access to this treatment. However, this would require significant investment in training and infrastructure, as well as ongoing funding for the teams and facilities. The problem of supplies availability is closely related to the difficulties imposed by the importation system adopted by the Brazilian government, but some efforts have already been made by the Brazilian scientific community to change this scenario.

### Age

The third cause of pancreas refusal for islet transplantation was age (8.5%). As in most countries, in Brazil islet transplantation is considered as an experimental procedure, therefore, pancreata offered to our group had been previously refused by other whole organ pancreas procurement teams. According to the ABTO, in 2008, 38% of the heart-beating donors were between 41 and 60 years old [27], however, only 60% of the donors considered in the present study belong to this age range. This could reflect the low proportion of pancreata from young donors offered for islet transplantation, since organs from younger donors are more likely to be accepted for whole organ pancreas transplantation teams. Since the donor age seems to be directly related to the quality of isolated islets obtained [28], any action leading to a decrease in the potential donor mean age could improve the outcome of the islet isolation process.

### Positive serology

The fourth cause of pancreas refusal for islet transplantation was positive serology (8.4%). This seropositivity refers to Chagas disease, toxoplasmosis, rubella, syphilis, cytomegalovirus, hepatitis B and C and HIV. Unfortunately we can accept the organs from these donors called marginal donor (considered not to be an ideal donor), since they present additional risk factors. The use of a marginal donor is only justified in situations in which the risk of patient death due to heart disease is greater than that offered by the donor [29].

### Hyperamylasemia

The fifth cause of pancreas refusal for islet transplantation was hyperamylasemia (8.2%). The serum amylase concentration reflects the balance between the rates of amylase entry into the bloodstream and its removal. Both the pancreas and the salivary glands display amylase concentrations which are several orders of magnitude greater than those displayed by any other normal tissue, with these two organs accounting for almost all of the serum amylase activity in normal individuals [30]. However, Albo and cols. [31] reported significant elevation in amylase serum levels after 24 h of patient admission, similarly to those patients admitted for cranial trauma. Another study showed persistent elevation in pancreatic enzymes in 19% of patients with isolated cranial trauma [32]. Vitale and cols*.* [33] showed that 38% of patients with severe head injury had increased total serum amylase levels with no evidence of acute pancreatitis. Moreover, the exocrine pancreatic enzyme production could be activated through central pathways in cases of head trauma, including increased vagal tone, release of activating hormones such as cholecystokinin and/or altered adrenergic stimulation [34]. The amylasemia reported in these previous studies reached values near 200U/L. If the amylasemia levels reach values above 400U/L, we refuse the pancreas for islet isolation and transplantation. Hyperamylasemia is one of the top five refusal causes (8.2%), since our analysis shows elevated amylasemia in 26% of the offered pancreata.

Most of the cases of brain death in Brazil are caused by violent death, correlating with head and abdominal traumas [35], which, in several cases, may elevate amylasemia. Other causes, such as: acute pancreatitis, pancreatic tumor and abscess indicate a lesion in this organ, which could lead to hyperamylasemia [30]. Therefore, under these situations, it is not possible to suggest actions to improve the pancreas conditions and, consequently, to diminish the amylasemia.

It is important to note that the cost of islets isolation and transplantation is an important issue in Brazil. The process of isolating and transplanting islets from deceased donors requires a specialized team with specific skills and knowledge, as well as specialized equipment and facilities. In Brazil, only three centers are available for this procedure, (1) our group NUCEL [8] which performed the first islet transplantation in Brazil in 2002 [9]; (2) a laboratory in Curitiba (Paraná), associated with the PUC-Paraná University and the Pro-Kidney Foundation, which conducted one islet transplant in 2005 [36], and, (3) a human islet isolation laboratory at the Endocrine Division of the Hospital de Clínicas de Porto Alegre – Rio Grande do Sul [37], but this unit has not yet transplanted islets into patients; leading to logistical challenges and increased costs for organ procurement and for patients who live far away from these centers.

Moreover, it is important to highlight that expanding organ procurement and transplantation services would also require significant investment in infrastructure, equipment and personnel, as well as ongoing funding and support. Additionally, there may be ethical and cultural barriers to organ donation and transplantation that need to be addressed in order to fully realize the potential benefits of these services.

## Conclusions

One of the major obstacles in organ transplantation, in general, is the limited supply of organs in adequate conditions for transplantation. Despite all progress in medical biotechnology, tissue engineering and stem cell research, in many cases, the only way to treat the patient is organ transplantation, which is not feasible without organ donation. Hopefully, in the near future, specialized cells and organs will be generated in vitro, rendering organ donation no longer an issue. Until then, it is important to find ways to perfect the organ donation system in order to viabilize treatment for a larger number of patients.

Considering the pancreas offers for islet transplantation in Brazil, our study points to the need to concentrate the efforts in actions which allow improvements in the quality of the donors´ maintenance at the ICU and minimize the technical issues. We believe that the conclusions shown in this work could help to increase the availability of better quality pancreas, leading to improvement of the islet isolation and transplantation outcome.

## Data Availability

The datasets used and analyzed during the current study are available from the corresponding author on reasonable request.

## Abbreviations

ANVISA: Brazilian Regulatory Agency
BMI: Body mass index
NUCEL: Cell and Molecular Therapy Center
cGMP: Current Good Manufacturing Practices
Hospital das Clínicas: Ethics Committee for Analysis of Research Projects
CAPPesq: Faculdade de Medicina Universidade de São Paulo
HIV: Human immunodeficiency virus
ICU: Intensive Care Unit
iEQs: Islet equivalents
CONEP: National Research Ethics Committee
pmp: Part per million
RDC: Resolution of The Collegiate Board
NTS: The National Transplant System
SST: The State System of Transplantation
ABTO: The Brazilian Association for Organ Transplant
SBD: The Brazilian Diabetes Society
TC: The Transplantation Center
SPOTs: The Organ and Tissues Procurement Organization Service
T1D: Type 1 diabetes mellitus
